# Resveratrol synthase homologs participate in infection of *Nicotiana benthamiana* by pathogenic plant viruses and fungi

**DOI:** 10.3389/fmicb.2025.1534785

**Published:** 2025-04-02

**Authors:** Zhuo Meng, Haijuan Wang, Guozhi Guo, Junxia Miao, Juan Liu, Hongyou Zhou, Mingmin Zhao, Baozhu Dong

**Affiliations:** ^1^College of Horticulture and Plant Protection, Inner Mongolia Agricultural University, Hohhot, China; ^2^Green Industry Development Center, Wuyuan, China; ^3^Ordos Wantong Agriculture and Animal Husbandry Technology Co., Ltd., Dalad Banner, China; ^4^Key Laboratory of the Development and Resource Utilization of Biological Pesticide in Inner Mongolia, Hohhot, China

**Keywords:** resveratrol synthase, *Nicotiana benthamiana*, turnip mosaic virus, *Botrytis cinerea*, *Arachis hypogaea* L

## Abstract

**Introduction:**

Resveratrol synthase (RS) is a key enzyme involved in the synthesis of stilbene and resveratrol. Resveratrol has many biological pharmacological activities that are beneficial to human health including anti-cancer, cardiovascular protection, estrogen regulation, antibacterial, antiviral, and reduction of tissue and organ damage. In plants, RS catalyzes the production of resveratrol, which helps to protect against fungal and bacterial diseases.

**Methods:**

We analyzed RS homologues from peanuts (*Arachis hypogaea* L.) during infection by plant viruses and fungi. The peanut RS gene was cloned and characterized. The peanut RS gene was cloned into the pEAQ-HT-DEST3 plant binary expression vector and transiently expressed in *Nicotiana benthamiana*.

**Results:**

Sequence analysis of the protein revealed a conserved stilbene synthase activity domain. The protein displayed high phylogenetic identity to RS from *A. hypogaea* (100%), *Vitis vinifera* (72.42%), and *Polygonum cuspidatum* (69.51). The results showed that RS expression in plants significantly contributed to infection by turnip mosaic virus (TuMV) and silghtly contributed to viral infection of tobacco mosaic virus (TMV). However, no significant influence of RS expression on infection by tobacco vein mottling virus (TVMV) was observed. Expression of the RS gene was transiently increased upon fungal infection of *Botrytis cinerea* in *N. benthamiana*.

**Discussion:**

This finding suggests that transient expression of the RS gene could significantly contribute to infection by turnip mosaic virus (TuMV) and improve the resistance of *N. benthamiana* to *B. cinerea*.

## 1 Introduction

The variety of biological properties of resveratrol includes anti-tumor, anti-oxidation (Silva et al., 2019), anti-free radical (Lee et al., 2010), anti-inflammatory (Chen et al., 2022; Prakash et al., 2024), antibacterial, anti-cancer (Rauf et al., 2018; Ren et al., 2021) activities, and cardiovascular protection (Breuss et al., 2019). Resveratrol is used as an additive in drugs, alcohol, or cosmetics. Beauty and health care products containing resveratrol are purported to delay aging, maintain skin moisture, and remove sores and chloasma (Ratz-Łyko and Arct, 2019; Zhou et al., 2021; Kumar et al., 2024). In animals, dietary supplementation with resveratrol has beneficial effects on health and improves immunity and production performance (Zhou et al., 2022). The efficacy of resveratrol combined with other antiviral drugs in inhibiting viral infection may permit the reduction or even elimination of latent virus (Wang et al., 2002). Resveratrol can inhibit viral replication. For example, resveratrol inhibits the reverse transcriptase of human immunodeficiency virus (HIV) and prevents HIV viral recovery, thereby enhancing sensitivity to antiviral drugs (Yang et al., 2006).

Resveratrol is a non-flavonoid polyphenol that contains a stilbene structure. Closely related to plant disease resistance (Kiselev and Dubrovina, 2021). RS is the last key enzyme in the resveratrol biosynthesis pathway and is the only necessary synthase in this pathway (Rolfs et al., 1981). Resveratrol is mainly synthesized via the phenylalanine metabolic pathway. The most critical regulatory enzyme in this pathway is RS (also known as 3-trihydroxystilbene synthase or stilbene synthase, STS) (Delaunois et al., 2009). Cloned STS genes can be divided into two types. One type is RS, which uses coumaryl-CoA and malonyl-CoA as specific substrates (Lanz et al., 1990). The other type is pinosylvin synthase (PS), which uses cinnamyl-CoA and malonyl-CoA as specific substrates to synthesize pinosylvin (Schwekendiek et al., 1992). Many inducible STS genes, such as RS, have been cloned and identified in peanut, pine, grape, and other plants (Zheng et al., 2014). Studies have shown that it is necessary to synthesize a stilbene compound-resveratrol-related substrate that plays the role as phytoalexin (Kiselev and Dubrovina, 2021). In the presence of the substrate, RS catalyzes the formation of stilbenes. Although there are enzyme substrates in almost all higher plants in nature, few RS genes can synthesize these substances under the action of inducing factors (Akiyama et al., 1999).

RS is produced by plants experiencing biotic stresses that include bacterial or fungal infection, or biotic stresses such as exposure to ultraviolet radiation, shock, and others (Sales and Resurreccion, 2014). RS highly accumulates in more than 70 types of plants, particularly grape (*Vitis vinifera*), peanut (*Arachis hypogaea* L.), and Japanese knotweed (*Polygonum cuspidatum*) (Jeandet et al., 2010; Dao et al., 2011). At present, the RS gene was cloned from peanut and then transgenicized into *Rehmannia glutinosa* (Lim et al., 2005), potatoes (*Solanum tuberosum* L.) (Yi et al., 2009), rice (Zheng et al., 2015), purple sweet potato (Pan et al., 2012), *Astragalus membranaceus* (Sun et al., 2017), jujube (Luo et al., 2015), and *Salvia miltiorrhiza*(Chen et al., 2007) can be stably and successfully expressed. Using the plant transient expression system, the peanut RS genes were successfully expressed in *N. benthamiana* (Condori et al., 2009), and the RS genes in knotweed and grapes using the silkworm- baculovirus expression vectors system (Pan et al., 2014). Overexpressing fusion proteins of 4-coumaroyl-CoA ligase (4CL) and stilbene synthase (STS) in tobacco plants leading to resveratrol accumulation and improved stress tolerance (He et al., 2018).RS activity has also been closely related to disease resistance in plants (Kiselev and Dubrovina, 2021). Some grape and pine varieties, legumes, and other plants can induce the synthesis and accumulation of stilbene compounds in injured tissues during infection by pathogens, which can inhibit further infection (Sparvoli et al., 1994; Zernova et al., 2014; Kiselev et al., 2016; Liu et al., 2019). RS genes from different sources have been transformed into tobacco (Hain et al., 1990), tomato (Giovinazzo et al., 2005), rice (Stark-Lorenzen et al., 1997; Leckband and Lörz, 1998), and alfalfa (Hipskind and Paiva, 2000). The transient expression of foreign RS gene in *Nicotiana benthamiana* has been reported (Condori et al., 2009). In summary, there are many reports on the study of RS gene resistance to pathogens in peanut, which was also confirmed in this experiment, but the analysis of RS gene protein structure and the effect of RS gene on plant virus infection have not been reported.

In the present study, the RS gene was amplified from peanuts using PCR. The gene was inserted into the pEAQ-HT-DEST3 plant binary expression vector using the Gateway recombinant system to create pEAQ-RS. To evaluate the role of the RS gene in plant viral infections, *Agrobacterium tumefaciens* harboring pEAQ-RS was used to transform *N. benthamiana* leaves, followed by inoculation of the leaves with either plant viruses tobacco vein mottling virus (TVMV), tobacco mosaic virus (TMV), or turnip mosaic virus (TuMV). The effect of RS overexpression on the resistance of *N. benthamiana* to *Botrytis cinerea* was also studied.It is of great significance to study the expression mechanism and process of RS gene, which improves plant disease resistance and can provide a theoretical premise and foundation for obtaining excellent disease-resistant varieties, and also solves the problems of shortage of resveratrol resources, more difficult to extract and isolate, and higher extraction cost.

## 2 Materials and methods

### 2.1 Experimental materials

PDONR207 and pEAQ-HT-DEST3 expression vectors, and *Agrobacterium tumefaciens* C58C1 were provided by Professor Juan Antonio García (Centro Nacional de Biotecnologia, CSIC, Spain). The infection clone of TVMV, TMV, TuMV was described by Zhao et al. (2020).

### 2.2 Plant preparation

Seeds of *N. benthamiana* were preserved in our laboratory and grown in a greenhouse at 22°C with alternating 16 h light and 8 h dark periods. The instantaneous infection experiment was used when the seedlings grew to the stage of 4–5 leaves and growth was the same.

### 2.3 Cloning of RS gene

Gene-specific primers were designed based on the RS sequence (AY170347) (Supplementary Table S1). The 50 μL reaction mixture for PCR included 10X PCR buffer, 5 μL dNTP mixture, 4 μL upstream primer, 2 μL downstream primer, 2 μL peanut cDNA 1 μL and Taq 0.25 μL, with deionized distilled water added to produce a final volume of 50 μL PCR included cycles were denaturation at 95°C for 5 min. 29 cycles of denaturation at 95°C, annealing at 70°C, annealing at 70°C, and extension at 72°C for 2 min. and a final extension at 72°C for 10 min. The amplified products were subjected to 1% agarose gel electrophoresis. PCR fragments containing the peanut RS gene (1,228 bp) were recovered and purified using an Agarose gel DNA recovery kit (TIANGEN, China). The gel recovery product was recombined with the pDONR207 intermediate vector by BP reaction and incubated overnight at 25°C. All the reaction products were transformed into *Escherichia coli* DH5α competent cells by heat shock, and then plated on LB solid medium containing gentamycin and grown overnight at 37°C. Bacterial clones were selected, propagated in LB broth containing gentamycin for 8–12 h, and verified by PCR. The pDONR207-RS plasmid was extracted and verified using *Bgl*II and *Pst*I (3,329 and 1,212 bp, respectively). The RS gene fragment was cloned into the pEAQ-HT-DEST3 binary expression vector using the LR reaction system (pDONR207-RS, 155.2 ng/μL), 3.5 μL pEAQ-HT-DEST3 (74.1 ng/μL) and (LR Clonase™ II enzyme mix, 1 μL) at 25°C for 8.5 h. Reaction products were transformed into *E. coli* DH5α competent cells by heat shock and incubated on LB solid medium containing kanamycin. After overnight culture at 37°C, the clones were selected and propagated in LB liquid medium containing kanamycin for 12 h. Positive clones of pEAQ-RS were verified by PCR and restriction enzyme digestion using *Spe*I and *ApaL*I.

### 2.4 *In silico* sequence and structure analysis

Sequences were aligned using ClustalW and SnapGene, and the evolutionary history was inferred using MEGA11 (Tamura et al., 2021), the genetic relationship of RS nucleotide level in different plants was explored. *In silico* analyses of the physicochemical properties, secondary structures, and conserved domains of the RS proteins were performed using ExPaSy, ProtParam, and NovoPro, respectively. Three-dimensional structural models of RS were predicted using Swiss-Model (Waterhouse et al., 2018). Conserved domains of *Bos taurus* (NP_001068604.1),^1^ *Rattus norvegicus* (NP_620806.2),^2^ *Acinetobacter* (RJE70264.1),^3^ *Alternaria* (ANW48382.1),^4^ *Polygonum cuspidatum* (ABI78940.1),^5^ and *Vitis vinifera* (XP_002272129.2),^6^ proteins were downloaded from NCBI.^7^ The structural similarity matrix between *A. hypogaea* RS and different species was obtained by TBtools (Chen et al., 2020). The structural tree diagram between *A. hypogaea* RS and different species was obtained using MEGA11 software.

### 2.5 Transient expression of RS in *Nicotiana benthamiana*

The resulting pEAQ-RS constructs were transformed into *A. tumefaciens* C58C1 using the freeze–thaw method. Bacteria harboring the pEAQ-RS plasmid were cultured overnight at 28°C and centrifuged at 4000 rpm for 15 min. The supernatant was discarded, and bacterial pellets were suspended to an optical density at 600 nm (OD_600_) = 1 in buffer containing 0.5 M 2-(N-morpholino)ethanesulfonic acid hydrate (pH 5.6), 1 M MgCl_2_, and 0.1 M acetosyringone. Each bacterial suspension was incubated at room temperature for 5 h and then used to infiltrate leaves of 4-5-week-old *N. benthamiana* plants using a needleless syringe. Leaf samples infiltrated with empty pEAQ-HT-DEST3 vector were used as controls. Twelve plants were used per treatment for each experiment.

### 2.6 Reverse transcription-quantitative PCR

Normally, the gene expression reached to the highest level at 3 days post infiltration in transient expression assay. Thus, we had chosen the samples from the inoculated leaves at 3dpi.Total RNA purified from *A. hypogaea* with 4–5 age leaves and was used for cDNA synthesis reactions. The cDNA was used for PCR performed with MonAmp™ SYBR Green qPCR mix (Monad, China) and gene-specific primers (Supplementary Table S1) in a model FTC-3000P Real-Time Quantitative Thermal Cycler (Funglyn Biotech, Canada). Expression was normalized using NbUBI as a reference. Fold changes relative to the control condition were calculated by the ΔΔ^CT^ method (Yue et al., 2022). In order to clarify the differences between samples, we have used SPSS software for significance analysis.

### 2.7 Co-agroinfiltration of pEAQ-RS and viral infection clone

Agrobacteria carrying pEAQ-RS, pEAQ-HT-DEST3, pLX-TVMV, TMV-green fluorescent protein (GFP), and TuMV-GFP were cultured and grown to OD_600_ = 1.0. Mixtures of pEAQ-RS/TVMV, pEAQ-RS/TMV-GFP, and pEAQ-RS/TuMV-GFP were prepared (3:1, v/v). Healthy plants and plants co-infiltrated with buffer or pEAQ-HT-DEST3 containing each virus (3:1, v/v) were used as controls. At 5 to 11 days post-infiltration, plant symptoms of different viruses were observed and photographed.

### 2.8 Western blot detection of virus proteins

Detection of RS proteins in complex samples based on specific binding of antigen and antibody. Total protein extracts from the plant samples were isolated and resolved by 12% sodium dodecyl sulfate-polyacrylamide gel electrophoresis. The protein gel was photographed using the Geldoc system (Thermo Fisher Scientific, United States) for loading control. Proteins were electroblotted onto nitrocellulose membranes as previously described by Degasperi et al. (2014). Immunodetection was conducted using primary antibodies, including anti-His tag antibody for RS, anti-coat protein (CP) for TVMV, and anti-GFP antibody for TuMV and TMV. Horseradish peroxidase-conjugated goat anti-rabbit IgG (ab205718; Abcam, United Kingdom) and mouse monoclonal antibodies were used as secondary antibodies. Protein signals were visualized by enhanced chemiluminescence. Quantification values from immunoblotting assays were normalized by the sum of the replicate approach.

To further confirm the RS contributing TuMV infection, we have performed the kinetic analysis with different dilution of RS plus TuMV infection clone in transient expression assay. After preparing the agrobacteria culture carrying RS and TuMV infection clone with OD_600_ = 1, the dilution ratios of RS and TuMV (v:v) were conducted as 0.5:1,1:1, 2:1 and 3:1. The TuMV infection clone was co-infiltrated with empty vector pEAQ-HT-DEST3 and Buffer as the control. The healthy plants were used as the negative control. The plants with 4–5 leaves were subjected for co-infiltrated assay with above dilutions. At 7 dpi, the upper leaves were collected and used for viral accumulation analysis by wester blot.

### 2.9 qPCR detection of key genes related with salicylic acid and jasmonic acid pathways in plants co-infiltration of RS and viruses

The expression of several key genes in salicylic acid (SA) and jasmonic acid (JA) pathways were subjected for Quantitative PCR. We have selected 3 genes (NtPAL, NtNPR1, NtICS) from SA pathway and 3 genes (NtCOI1, NtLOX1, NtAOS) from JA pathway. The sequeces of these genes were searched from database of *N. benthamiana* in solgenomic.^8^ The primers used for qPCR assay NtCOI1-F/NtCOI1-R, NtLOX1-F/NtLOX1-R, NtAOS-F/NtAOS-R, NtNPR1-F/NtNPR1-R, NtICS-F/NtICS-R, NtPAL-F/NtPAL-R, and NtUB1-F/NtUB1-R were designed and synthesized by Sangyo Bioengineering (Shanghai) Co. (Supplementary Table S1). The expression level of these genes was determined by qRT-PCR in samples of co-expression RS and different viruses (TuMV,TVMV and TMV). Three biological replicates and three technical replicates per treatment were designed.

### 2.10 Transient expression of RS gene during *Botrytis* *cinerea* infection

*Botrytis cinerea* was inoculated on PDA medium for subculture activation culture at 25°C for 7 days. The spore suspension was diluted to 10^5^ CFU/mL. *N.benthamiana* plants were infiltrated with pEAQ-RS, 3 days later, the spore suspension of *B. cinerea* (50 μL)were lightly dropped onto the needle-scratched leaves. After incubation in the dark for 24 h, the leaves were grown using alternating cycles of light and dark (16:8 h). The lesion area was measured using ImageJ software (NIH, United States). All experiments were repeated three times.

## 3 Results

### 3.1 Cloning of RS homologs from peanut

**Figure 1:**
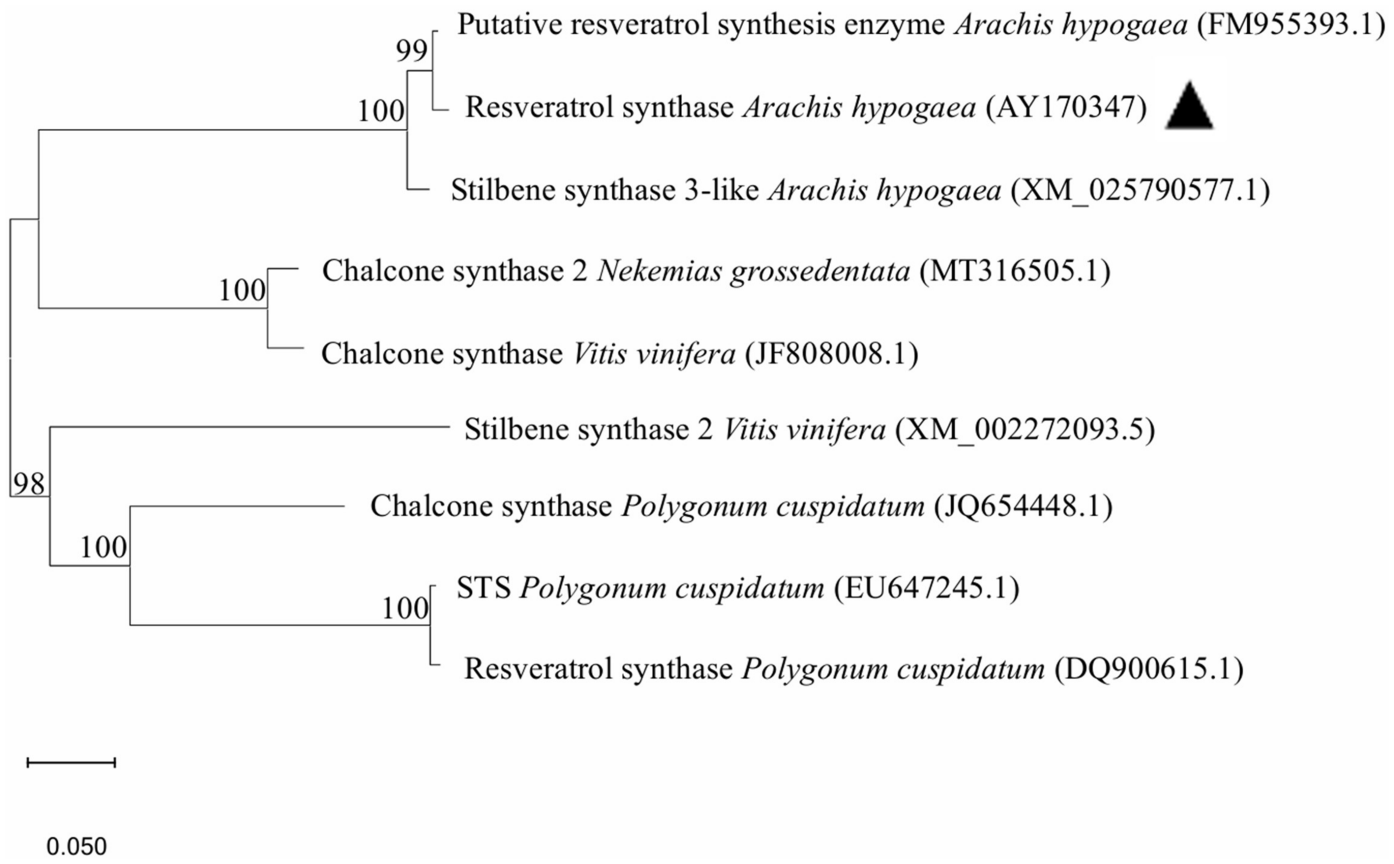
Phylogenetic analysis of RS. The phylogenetic tree was constructed based on the RS sequences and chalcone synthase using the neighbor-joining method in MEGA software (version 11.0). The RS in peanut (*Arachis hypogaea*) is labeled with a black arrow.

Based on the RS gene sequence recorded in peanuts (*A. hypogaea*, AY170347), we designed primers to amplify RS transcripts (Supplementary Table S1). Using peanut cDNA as a template, a 1,228 bp band (1,167 bp of RS plus attB1 and attB2) was amplified by PCR (Supplementary Figure S1). The RS gene was cloned into pDONR207 using the BP recombination reaction to create pDONR-RS, which was sequenced (Supplementary Figures S2, S3). Specific bands of 3,329 and 1,212 bp were obtained by restriction endonuclease digestion using *Bgl*II and *Pst*I, respectively (Supplementary Figure S1). The RS gene in pDONR-RS was cloned into the pEAQ-HT-DEST3 plant binary vector using the LR reaction to obtain pEAQ-RS. The pEAQ-RS plasmid was verified using the restriction enzymes *Spe*I and *ApaL*I. respective bands of 7,895 and 3,304 bp were obtained (Supplementary Figure S1), indicating successful construction of the pEAQ-RS expression vector. The *A. hypogaea* RS gene was subjected to a BLAST search against public databases of *Nekemias grossedentata, Vitis vinifera*, and *Polygonum cuspidatum* (Figure 1). The *A. hypogaea* RS (AY170347) sequence was 100% identical to *P. cuspidatum* GenBank accession numbers EU647245.1 and DQ900615.1 at the nucleotide level. The identity of chalcone synthase from *Vitis vinifera* (JF808008.1) and *Nekemias grossedentata* (MT316505.1) with the *A. hypogaea* RS GenBank accession number AY170347 was as high as 100% at the nucleotide level. A distant cluster of stilbene synthase *Vitis vinifera* (XM_002272093.5) was 98% formed by the RS from *A. hypogaea*.

### 3.2 Peanut RS contains the STS active and signal sites

**Figure 2:**
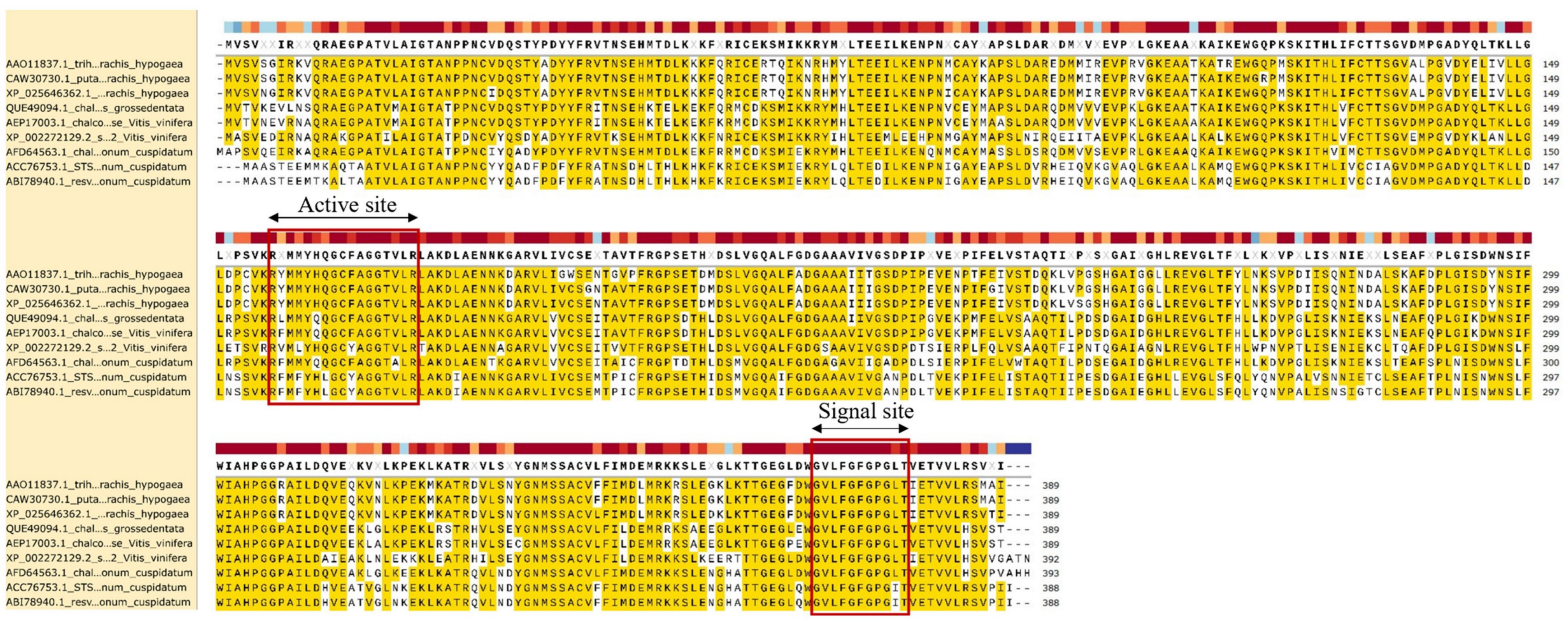
Protein sequence alignment of RS. Sequence conservation and consensus secondary structures (ClustalW and SnapGene) are shown for the structural superimposition. The red square highlights the stilbene synthase (STS) active site (RYMMYHQGCFAGGTVLR) and signal site (GVLFGFGPGLT), which are critical role in the resveratrol synthesis pathway.

Translation of the cloned sequences resulted in an RS of 389 amino acids (GenBank: AAO11837.1). The physicochemical properties of the protein was analyzed using the ProtParam webserver (Table 1). The amino acid sequence homology and percentages of amino acid content are shown in Table 2. RS has the molecular formula C_1900_H_3022_N_516_O_566_S_22_, an average molecular weight of 42 kDa, and an isoelectric point of 6.35. A protein sequence search against the (PROMALS3D) protein profile database revealed a significant match to the stilbene synthase active site (RYMMYHQGCFAGGTVLR) at positions 156–172 of RS and the signal site (GVLFGFGPGLT) at positions 368–378 of RS, which is thought to be involved in the synthesis of resveratrol (Figure 2).

**Table 1:** Physicochemical parameters of RS protein.

Parameter	RS
Molecular formula	C_1900_H_3022_N_516_O_566_S_22_
Molecular weight	42855.33
Theoretical isoelectric point	6.35
Instability index	33.54
Aliphatic coefficient	86.48
Total number of negatively charged residues (Asp+Glu)	45
Total number of positively charged residues (Arg + Lys)	43
Grand average of hydropathicity	−0.147

**Table 2:** Comparison of amino acid sequence homology in the coding region of resveratrol synthase gene between *Arachis hypogaea* and *Vitis vinifera* and *polygonum cuspidatum.*

Plant	Genetic accession number	Amino acid sequence homology (%)
*Arachis hypogaea*	AAO11837.1	100
*A. hypogaea*	CAW30730.1	97.17
*A. hypogaea*	XP_025646362	95.89
*Nekemias grossedentata*	QUE49094.1	72.42
*Vitis vinifera*	AEP17003.1	71.39
*V. vinifera*	XP_002272129.2	67.96
*Polygonum cuspidatum*	AFD64563.1	69.51
*P. cuspidatum*	ACC76753.1	63.35
*P. cuspidatum*	ABI78940.1	63.35

### 3.3 *Arachis hypogaea* RS is structurally related to *Vitis vinifera* and *Polygonum cuspidatum* RS

**Figure 3:**
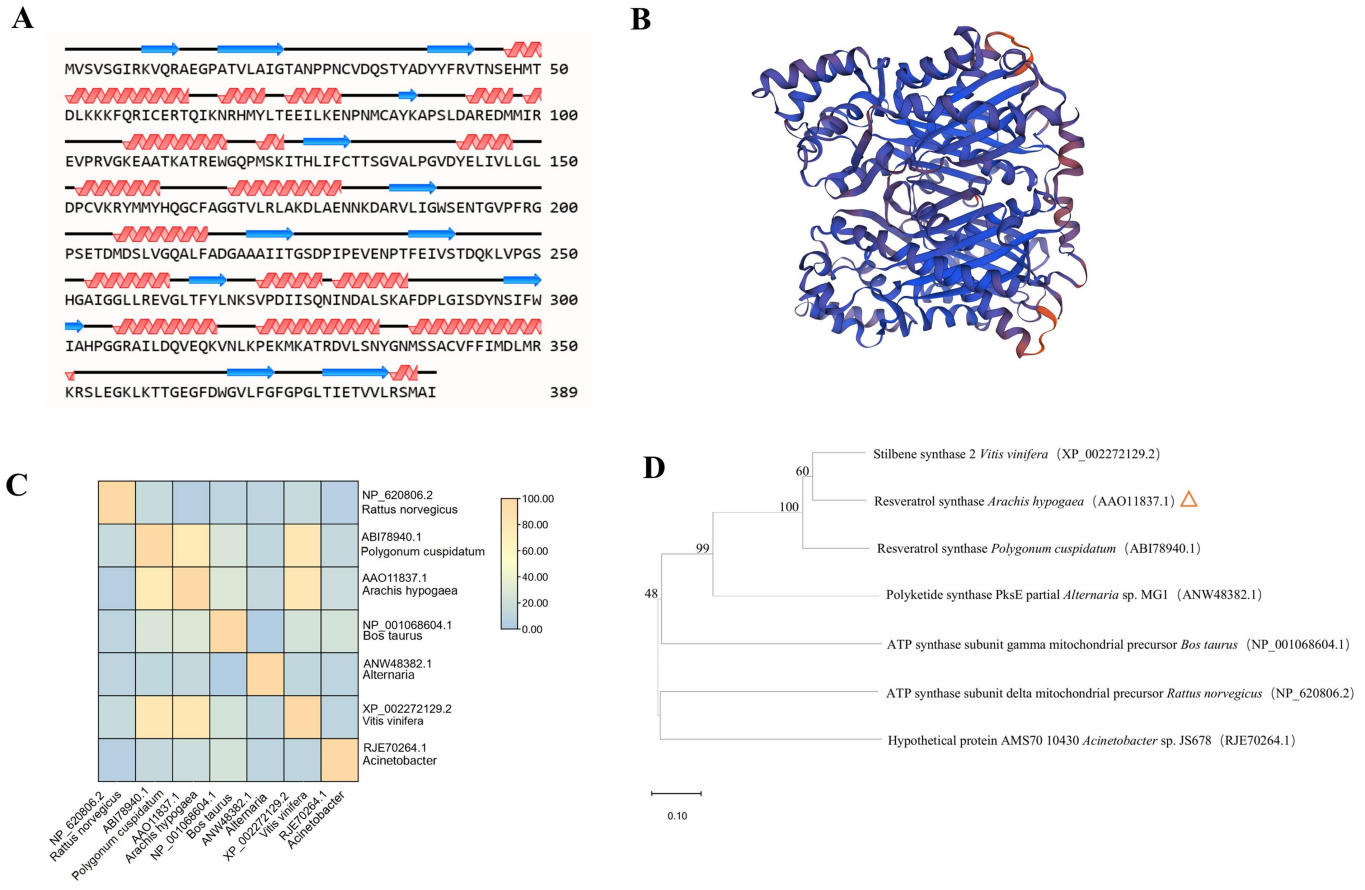
Structural modeling and analysis of RS. **(A)** Secondary structures derived from the predicted models of RS. **(B)** Tertiary structural models of RS are shown. Colors denote prediction confidence scores. **(C)** Structural similarity matrix between RS and different species. **(D)** The structural dendrogram of RS and different species. Branch support values are shown. *Rattus norvegicus*, *Polygonum cuspidatum*, *Bos taurus*, *Alternaria*, *Vitis vinifera*, *Acinetobacter* are from NCBI.

STS shares more sequence identity, and the active and signal sites are conserved across RS proteins. Recently developed deep-learning algorithms can predict high-resolution protein structures from primary sequences. The models obtained showed a conserved core with high prediction confidence scores, flanked by less conserved termini with a variable number of disordered residues (Figure 3; Supplementary Table S2).

The secondary structure of RS was analyzed using NovoPro (Figure 3A). In the figure, the helix represents an alpha helix, the arrow represents a random coil, and the remainder represents the extended strand. The alpha helix and random coil are the main structural elements of RS, and the extended strand is scattered throughout the protein. The three-dimensional structure of RS predicted by Swiss-Model obtained a tertiary structure model with a GMQE value of 0.90 (Figure 3B).

To confirm that the cloned *A. hypogaea* RS gene is structurally related to known RS enzymes, structural comparison and alignment analyses were performed with reference structures obtained by X-ray crystallography or high-resolution modeling. Structural similarity matrix and hierarchical clustering results indicated that *A. hypogaea* RS has spatial topologies close to those of *P. cuspidatum* RS and *Alternaria* PS, which differed significantly from those of the ATP synthase subunit delta mitochondrial precursor *Rattus norvegicus* and *Acinetobacter hypothetical* protein (Figures 3C,D).

### 3.4 Expression of RS contributes to TuMV infection in *Nicotiana benthamiana*

**Figure 4:**
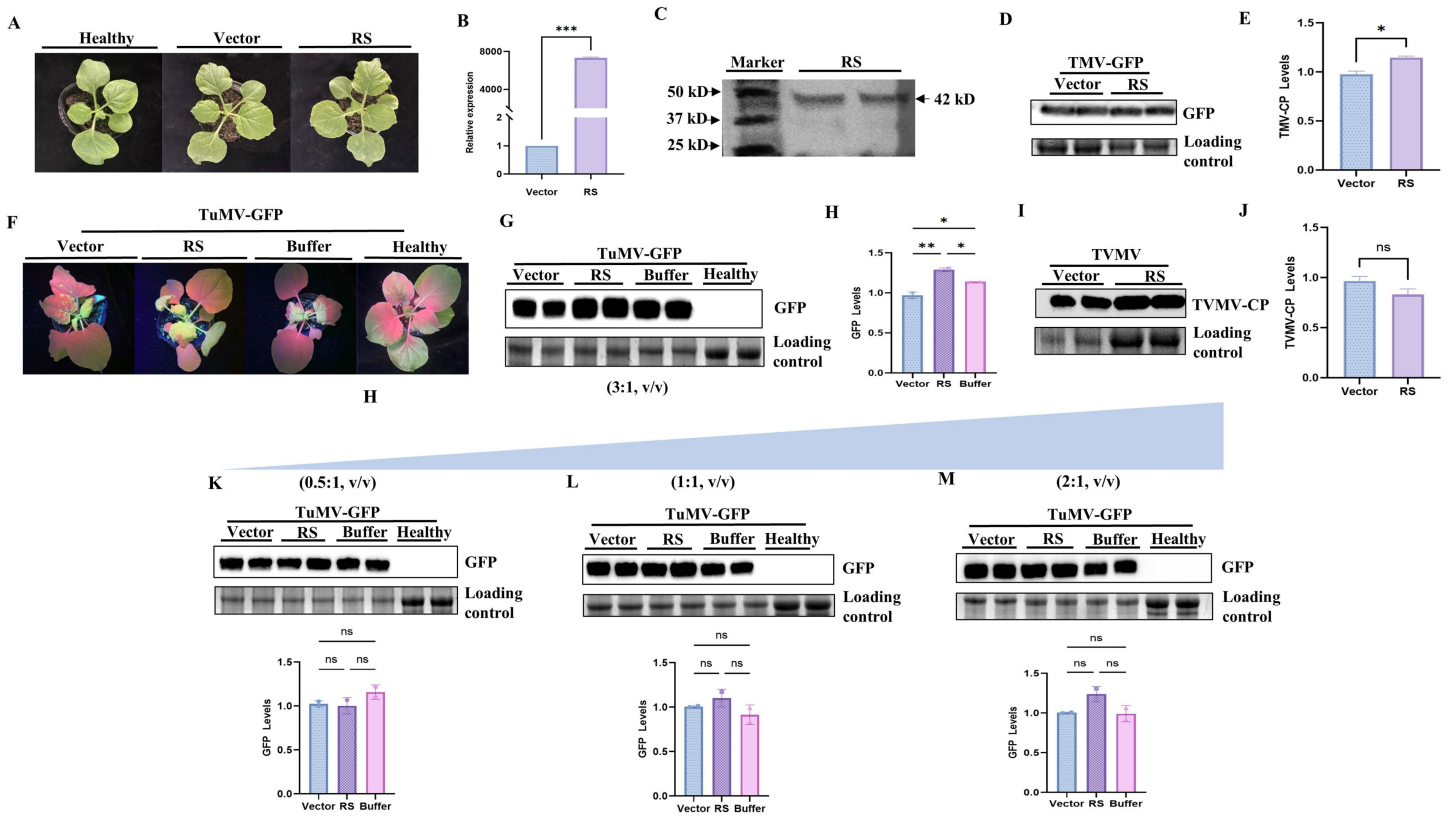
Expression analysis of RS and effect of transient expression of RS on TVMV, TMV, and TuMV virus infection. **(A)** Plant phenotype after transient expression of *Nicotiana benthamiana*. **(B)** qPCR detection of the expression of RS in agro-infiltrated leaves. *** indicates a significant difference between the data and the control. **(C)** Western blot analysis of RS expression. A 42 kD protein band was detected by western blot (anti-His). **(D)** Accumulation of TMV virus detected by GFP fluorescence in western blot. **(E)** Quantification of viral accumulation by Quantity ONE. **(F)** Symptoms of TuMV infection in *N. benthamiana* transiently expressing RS. **(G)** Accumulation of TuMV virus(3:1, v/v) detected by western blot (anti-GFP). **(H)** Quantification of viral accumulation by Quantity ONE. **(I)** Accumulation of TVMV virus detected by western blot (anti-CP). **(J)** Quantification of viral accumulation by Quantity ONE. **(K)** Accumulation of TuMV (0.5:1, v/v) virus detected by western blot (anti-GFP) and quantification of viral accumulation by Quantity ONE. **(L)** Accumulation of TuMV (1:1, v/v) virus detected by western blot (anti-GFP) and quantification of viral accumulation by Quantity ONE. **(M)** Accumulation of TuMV (2:1, v/v) virus detected by western blot (anti-GFP) and quantification of viral accumulation by Quantity ONE. ns, no significant difference between the data and the control, * slight difference between sample and control (*p* < 0.05), ** significant difference between sample and control (*p* < 0.01). ***Highly significant difference between sample and control (*p* < 0.001).

To verify the influence of RS protein on viral infection, overexpression constructs were used in transient expression assays. The pEAQ-HT-DEST3-RS expression vector was transformed into *Agrobacterium* strains that were subsequently infiltrated into *N. benthamiana* leaves. There were no significant differences in plant phenotype (Figure 4A). Specific bands for RS in *N. benthamiana* were obtained using RT-PCR (Supplementary Figure S3). Correspondingly, high expression levels of RS were detected using qPCR (Figure 4B). Protein expression of RS (42 kDa) in *N. benthamiana* was detected by western blot analysis, indicating that the overexpression constructs were functional (Figure 4C).

Co-infiltration of pEAQ-HT-DEST3-RS- and TuMV-GFP-infected clones in *N. benthamiana* was performed. Symptoms of TuMV infestation were most pronounced at 7d, the GFP fluorescence in the upper non-inoculated leaves was measured and photographed. Compared with the control, plants treated with RS showed an increase in GFP fluorescence (Figure 4F). The significant increasement of GFP accumulation were detected in plants co-infiltrated with RS and TuMV-GFP compared with the control (*p* < 0.01; Figures 4G,H). The accumulation of TMV in *N. benthamiana* co-injected with pEAQ-RS and TMV was slightly different from that of the control (*p* < 0.05; Figures 4D,E; Supplementary Figure S4). However, plants co-infiltrated with RS and TVMV did not show obvious differences in viral symptoms (Supplementary Figure S4) or viral accumulation detected by western blotting with anti-CP compared with those of control plants (Figures 4I,J; Supplementary Figure S4). The results reveal that overexpression of the RS homolog specifically contributes to TuMV resistance in *N. benthamiana*, but not to TVMV.

To further confirm the RS contributing TuMV infection, we have performed the kinetic analysis with different dilution of RS plus TuMV infection clone in transient expression assay. The results showed that there was no significant difference of viral the accumulation in samples co-infiltrated with pEAQ-RS and TuMV (0.5:1,1:1 and 2:1,V:V) and compared with the control (Figures 4K,L,M). However, the very significant difference of viral accumulation was observed in those samples co-infiltrated with pEAQ-RS and TuMV(3:1, V:V; *p* < 0.01; Figures 4G,H). This indicates that the amount of RS might be the threshold to promote TuMV infection.

### 3.5 PAL in SA pathway and LOX1 in JA pathway are correlative with RS contributing TuMV infection

**Figure 5:**
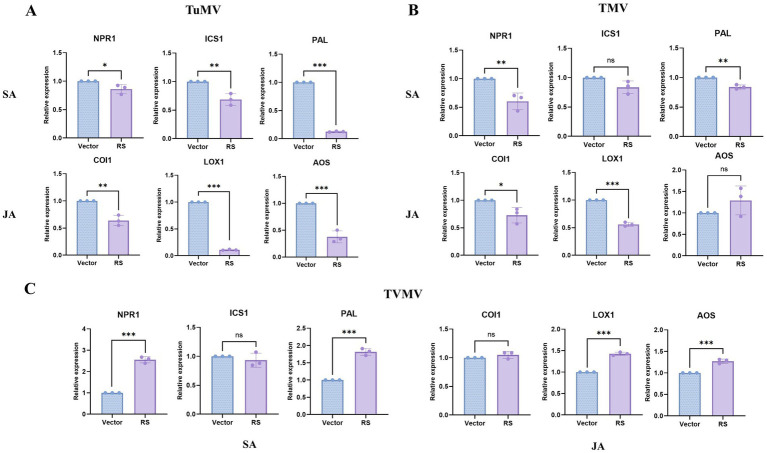
q-PCR detection of key genes related with SA and JA pathways in plants co-infiltration of RS and viruses **(A)** Key genes related with SA and JA pathways after TuMV infestation between sample and control. **(B)** Key genes related with SA and JA pathways after TMV infestation between sample and control. **(C)** Key genes related with SA and JA pathways after TVMV infestation between sample and control. *Slight difference between sample and control (*p* < 0.05), ** significant difference between sample and control (*p* < 0.01). ***Highly significant difference between sample and control (*p* < 0.001).

To explore the possible mechanism of RS contributing TuMV infection, we supposed that RS might influence the plant anti-viral resistance response. Thus, we have analyzed regulation role of RS in SA and JA pathways in plant.The results showed that the expression of NPR1, ICS1, PAL in the SA pathway, and COI1, LOX1, and AOS in the JA pathway decreased significantly after co-infiltration with RS and TuMV compared with that of the control. Particularly, PAL, LOX1, and AOS were decreasing to the most obvious extent (Figure 5A). In the case of co-infiltration with TMV, the expression of NPR1 and PAL in the SA pathway decreased significantly compared with the control. The expression of COI1 and LOX1 in the JA pathway decreased significantly (Figure 5B). After inoculation with TVMV, the expression of NPR1 and PAL in the SA pathway, and LOX1 and AOS in the JA pathway were significantly elevated compared with the control (Figure 5C). This is indicating that PAL in SA pathway and LOX1 in JA pathway are corelative with RS contributing TuMV infection. While the extent of decreasing level of these genes in TMV samples are slightly less than those of TuMV, which resulted in slightly contribution effect of RS on TMV infection.

### 3.6 Expression of RS inhibits *Botrytis cinerea* infection in *Nicotiana benthamiana*

**Figure 6:**
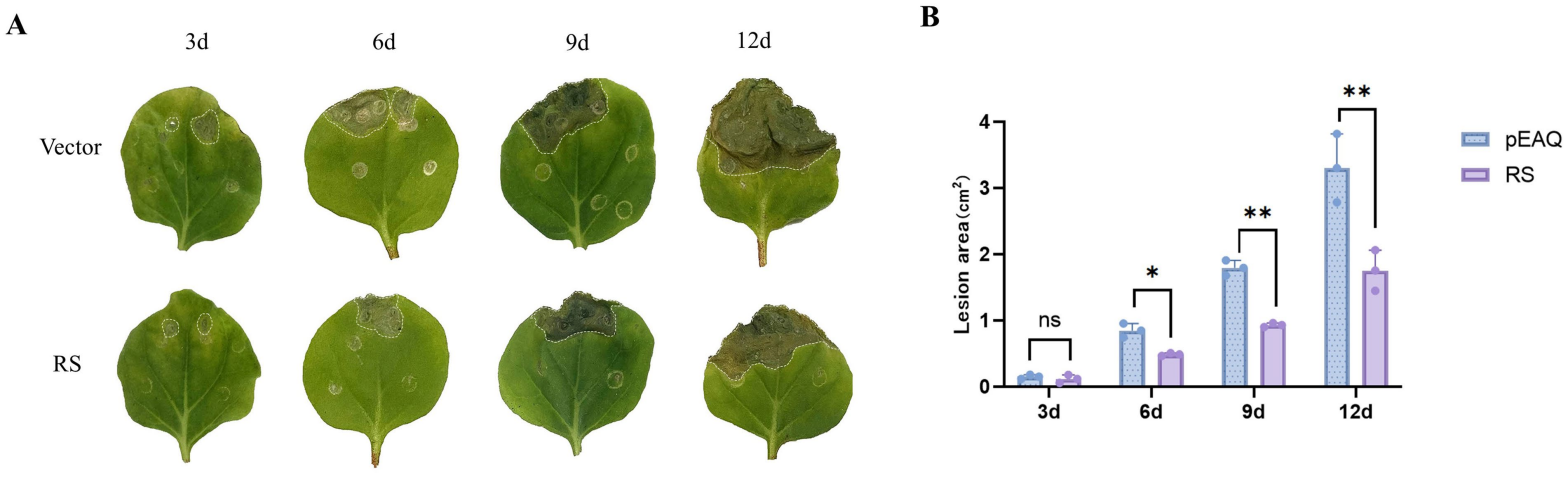
Effect of transient expression of RS gene on fungal infection with *Botrytis cinerea*. **(A)** Phenotypic changes at 3, 6, 9, and 12 d after inoculation with *B. cinerea*. **(B)** Necrotic spot area on *Nicotiana benthamiana* leaves inoculated with *B. cinerea*. **Significant difference between sample and control (*p* < 0.01).

To evaluate the involvement of the RS gene in fungal infection, *N. benthamiana* leaves were inoculated with *B. cinerea*. Phenotypic changes were observed at 3, 6, 9, and 12 d after inoculation (Figure 6A). The necrotic spot areas of the RS-treated leaves were smaller than those of the vector control. Particularly, at 9,12 d after inoculation with *B. cinerea,* the necrotic spot area of leaves was significantly decreased (*p* < 0.01). The findings suggest that activity of the RS gene regulates the resistance of *N. benthamiana* to *B. cinerea* (Figure 6B; Supplementary Table S3).

## 4 Discussion

As a phytoprotegerin, resveratrol has many beneficial effects on human health and animal production and can improve the resistance of plants to pathogens (Chang et al., 2011). Resveratrol potently inhibits a variety of viruses (Baur and Sinclair, 2006). The gene encoding RS has been found in *V. vinifera*, *A. hypogaea*, *P. cuspidatum*, and other plants. RS is a key enzyme in the synthesis of resveratrol and a necessary synthase in the synthesis process. RS belongs to a group of enzymes abbreviated STS, which is represented by a multigene family in most stilbenoid-producing species. STS occurs in a limited number of unrelated plants and synthesizes the backbone of stilbene phytoalexins, which have antifungal properties and contribute to pathogen defense (Lanz et al., 1991).

To date, studies on RS have involved the expression of multiple enzymes from the phenylpropanoid pathway in heterologous transgenic systems or by using stable transformations in transgenic plants (Zhang et al., 2006). RS transgenic kiwifruit, white poplar, and strawberry are reportedly more sensitive to fungal disease resistance (Kobayashi et al., 2000; Giorcelli et al., 2004; Hanhineva et al., 2009). The resistance of RS transgenic tobacco, tomato, and grape to *Phytophthora* and *B. cinerea* has improved (Hain et al., 1990; Adrian and Jeandet, 2012). The RS gene can increase the resistance to fungal and bacterial diseases in transgenic *N. benthamiana*. Trans-piceid (resveratrol glucoside) accumulates as the major stilbenoid compound in infected *N. benthamiana*. Activity of the RS gene is positively correlated with the ability to resist *B. cinerea* (Hain et al., 1993).

Transient expression of foreign RS genes and their effects on plant viral infections have rarely been reported. In this study, we cloned transcripts of *A. hypogaea* RS and characterized their gene sequences. In silico analyses confirmed the presence of the STS active site and signal site of RS. These data were supported by secondary and tertiary structure predictions and modeling. The N-terminal structure of *A. hypogaea* RS protein is similar to that of chalcone synthase (EC 2.3.1.74), naringenin chalcone synthase, STS, and claisen condensation synthase. A conserved cysteine residue is located in the central region of these proteins. Phylogenetic analysis showed that the cloned RS of *A. hypogaea* clustered with *P. cuspidatum* RS and *Alternaria* PS. These findings indicate that they may have similar activities.

We investigated their roles in plant viral infections. Plants expressing RS contributed to TuMV-GFP infection, followed by an increase in GFP fluorescence and TMV accumulation. However, no significant influence of RS expression on TVMV infection was observed. RS is the last key enzyme acting in the resveratrol anabolic pathway and the essential synthase in the synthesis pathway. Among them, resveratrol, as a secondary metabolite of astragalus, is synthesized mainly through the phenylalanine metabolic pathway (Delaunois et al., 2009). JA and SA are two important defense hormones that can assist plants in resisting pathogens (Ogawa et al., 2006; Jeong et al., 2016; Huang et al., 2020).PAL is a plant secondary metabolism, a key enzyme and rate-limiting enzyme of the phenylpropane pathway, which is directly related to plant disease resistance. When plants are infected by pathogens, usually PAL enzyme activity increases, along with the increase in the synthesis of antimicrobial substances such as lignin and chlorogenic acid, which act as chemical barriers in the process of plant disease resistance (Huang et al., 2010). Presumably, RS genes may play a negative regulatory role in the JA and SA hormone signaling pathways after plant virus infection. It is supposed that RS could inhibit the gene expression in SA pathway and JA pathway, which indirectly contribute viral infection. Next, the promotional effect of RS on TuMV could be further verified by interaction with TuMV-CP and subsequent gene locus mutations. From this point of view, this technology has a broad prospect and important significance, while the related research needs to be further strengthened. Transient expression of RS genes can improve the disease resistance of plants against *B. cinerea* and can promote the accumulation of TuMV and TMV infection. The effect on plant virus infestation can also be explored after modification of the chemical structure of RS (Song et al., 2021). However, if the RS gene is introduced into plants, whether the related secondary metabolites produced about stilbene compound influence beneficial microorganisms in plants, which may be beneficial or inhibit the growth of beneficial microorganisms. There are many substances and compounds that act as phytochemicals in plants, and whether the addition of the RS gene will influence the phytochemicals in the original plant. These questions have not yet been clearly and conclusively investigated, and further research can be conducted in the future.

Furthermore, transient expression of the RS gene inhibited *B. cinerea*. Therefore, exploring the potential applications of RS in bioengineering could provide an efficient way to improve disease resistance in plants and the nutritional quality of their fruits (He et al., 2018). The RS amount in transient expression assay is relatively limited and cannot be maintained for a long period of time (Steinbiss and Davidson, 1991). Therefore, it can only be applied to short-term mass expression of RS to provide technical validation. The resveratrol-rich plants can be utilized as plant bioreactors for mass expression of RS genes, which could reduce the corresponding time and cost problems for the synthesis of resveratrol (Kiselev and Dubrovina, 2021). Of course, when we want to obtain resistant plants, we can introduce the RS gene into important food and economic crops through transgenic or gene-editing technologies (Hain et al., 1990). Like this, the problem of difficult to obtain disease-resistant varieties of crops through conventional breeding methods can be effectively solved.Therefore, we expected that the leaf extract of RS expression plants could be applied as a new safe fungicide against different plant fungal diseases, which reduce crop loss with lower production cost. Our data also are providing a new insight to apply RS gene in the breeding of *B. cinerea* resistant plants.

## 5 Conclusion

This study provides novel insights into the regulatory function of Resveratrol synthase homologs on plant pathogen infection. Expression RS in plants significantly contributed to infection by TuMV and silghtly contributed to viral infection of TMV. RS also improved the resistance of *N. benthamiana* to fungal pathogen *B. cinerea*. This is suggestion that RS participates int. pathogen infection in plants”.

## Data Availability

The datasets presented in this study can be found in online repositories. The names of the repository/repositories and accession number(s) can be found in the article/Supplementary material.

## Footnotes

^1^ https://www.ncbi.nlm.nih.gov/protein/NP_001068604.1?report=genpept

^2^ https://www.ncbi.nlm.nih.gov/protein/NP_620806.2

^3^ https://www.ncbi.nlm.nih.gov/protein/RJE70264.1

^4^ https://www.ncbi.nlm.nih.gov/protein/ANW48382.1

^5^ https://www.ncbi.nlm.nih.gov/protein/ABI78940.1

^6^ https://www.ncbi.nlm.nih.gov/protein/XP_002272129.2

^7^ https://www.ncbi.nlm.nih.gov/

^8^ https://solgenomics.net/organism/Nicotiana_benthamiana/genome
