# Supplementary material for: Resveratrol synthase homologs participate in infection of Nicotiana benthamiana by pathogenic plant viruses and fungi
Source: Front Microbiol. 2025 Apr 2;16:1534785. doi: 10.3389/fmicb.2025.1534785 (PMC11999985; doi:10.3389/fmicb.2025.1534785)
Supplement: Supplementary file 1 [file Data_Sheet_1.docx]

**Figure S1.** Synthesis and cloning of RS. **(A)** Amplification of RS gene by PCR. Marker, 2 kb plus DNA Ladder. the 1228 bp fragment is shown. **(B)** pDONR-RS digestion. Marker, 2kb plus DNA Ladder. Products following digestion by *Bgl*Ⅱ and *Pst*I are shown in lane 1 and 2, respectively. **(C)** pEAQ-RS digestion. Marker, 15k DNA Ladder. Products following digestion of pEAQ-RS by *Spe*I and *ApaL*I are shown in lane 1 and 2, respectively


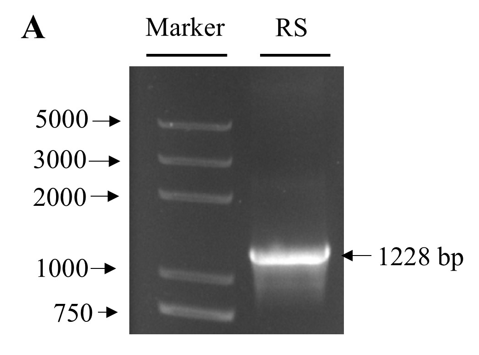

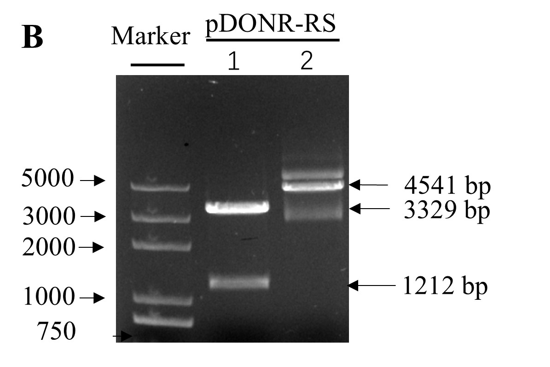


**
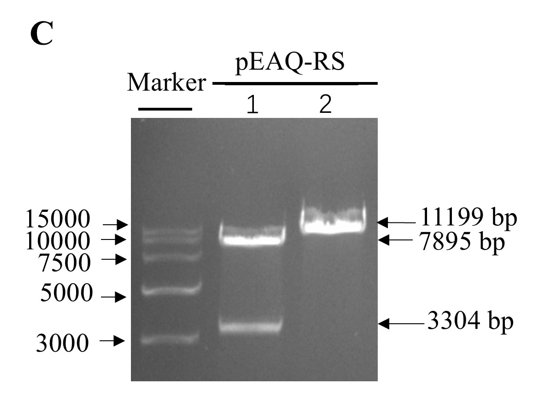
**

**Figure S2.** Sequence map of RS.


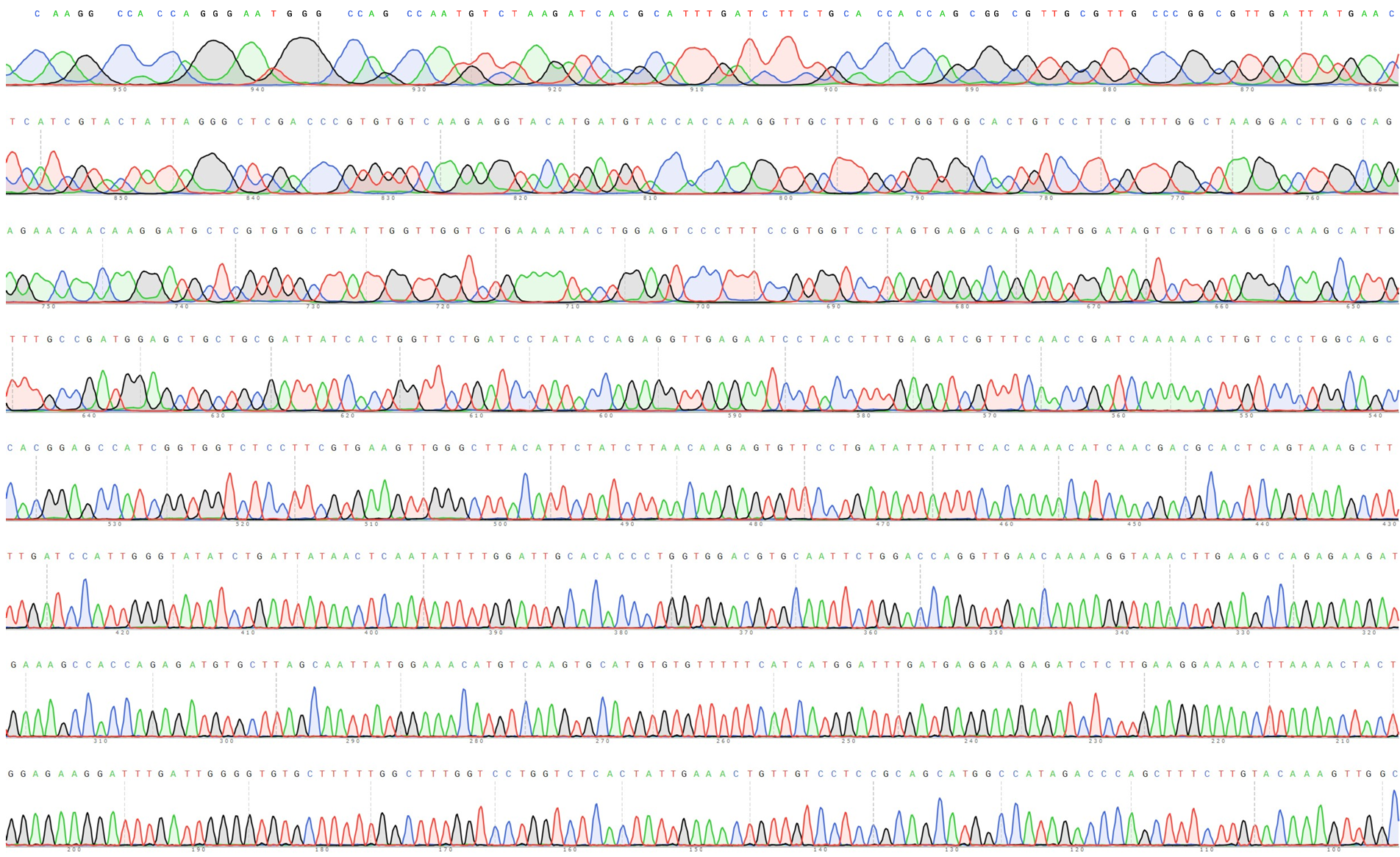

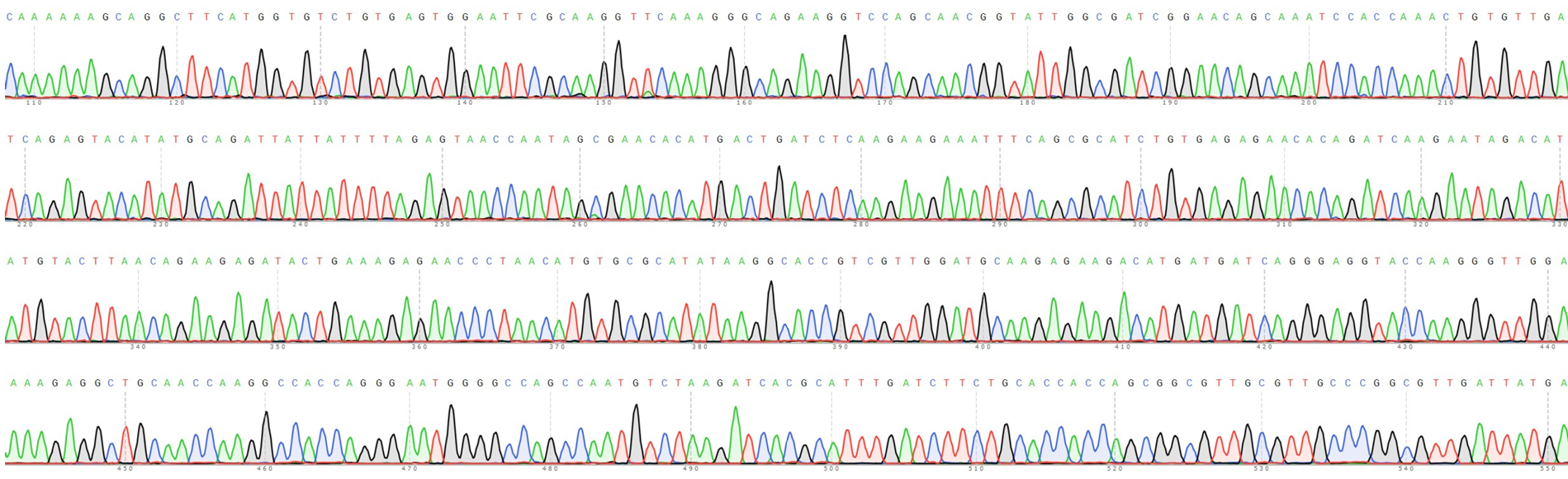


**Figure S3.** Sequence of RS.

5’ATGGTGTCTGTGAGTGGAATTCGCAAGGTTCAAAGGGCAGAAGGTCCAGCAACGGTATTGGCGATCGGAACAGCAAATCCACCAAACTGTGTTGATCAGAGTACATATGCAGATTATTATTTTAGAGTAACCAATAGCGAACACATGACTGATCTCAAGAAGAAATTTCAGCGCATCTGTGAGAGAACACAGATCAAGAATAGACATATGTACTTAACAGAAGAGATACTGAAAGAGAACCCTAACATGTGCGCATATAAGGCACCGTCGTTGGATGCAAGAGAAGACGTGATGATCAGGGAGGTACCAAGGGTTGGAAAAGAGGCTGCAACCAAGGCCACCAGGGAATGGGGCCAGCCAATGTCTAAGATCACGCATTTGATCTTCTGCACCACCAGCGGCGTTGCGTTGCCCGGCGTTGATTATGAACTCATCGTACTATTAGGGCTCGACCCGTGTGTCAAGAGGTACATGATGTACCACCAAGGTTGCTTTGCTGGTGGCACTGTCCTTCGTTTGGCTAAGGACTTGGCAGAGAACAACAAGGATGCTCGTGTGCTTATTGGTTGGTCTGAAAATACTGGAGTCCCTTTCCGTGGTCCTAGTGAGACAGATATGGATAGTCTTGTAGGGCAAGCATTGTTTGCCGATGGAGCTGCTGCGATTATCACTGGTTCTGATCCTATACCAGAGGTTGAGAATCCTACCTTTGAGATCGTTTCAACCGATCAAAAACTTGTCCCTGGCAGCCACGGAGCCATCGGTGGTCTCCTTCGTGAAGTTGGGCTTACATTCTATCTTAACAAGAGTGTTCCTGATATTATTTCACAAAACATCAACGACGCACTCAGTAAAGCTTTTGATCCATTGGGTATATCTGATTATAACTCAATATTTTGGATTGCACACCCTGGTGGACGTGCAATTCTGGACCAGGTTGAACAAAAGGTAAACTTGAAGCCAGAGAAGATGAAAGCCACCAGAGATGTGCTTAGCAATTATGGAAACATGTCAAGTGCATGTGTGTTTTTCATCATGGATTTGATGAGGAAGAGATCTCTTGAAGGAAAACTTAAAACTACTGGAGAAGGATTTGATTGGGGTGTGCTTTTTGGCTTTGGTCCTGGTCTCACTATTGAAACTGTTGTCCTCCGCAGCATGGCCATA 3’

**Figure S4.** Specific band of RS gene amplified by RT-PCR from infiltrated *N. benthamiana*.


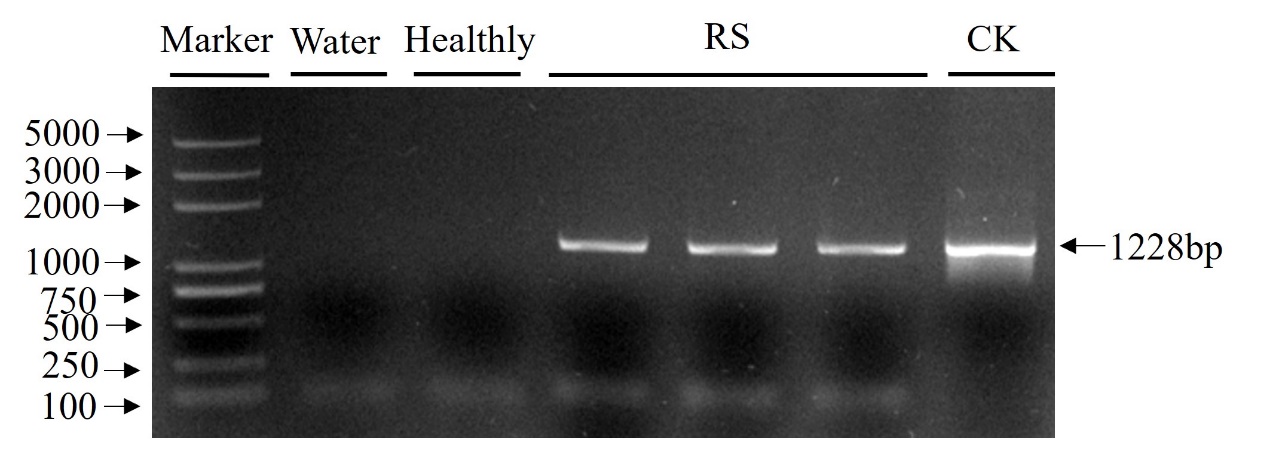


**Figure S5.** Viral symptoms of TVMV and TMV in RS treated plants.

**
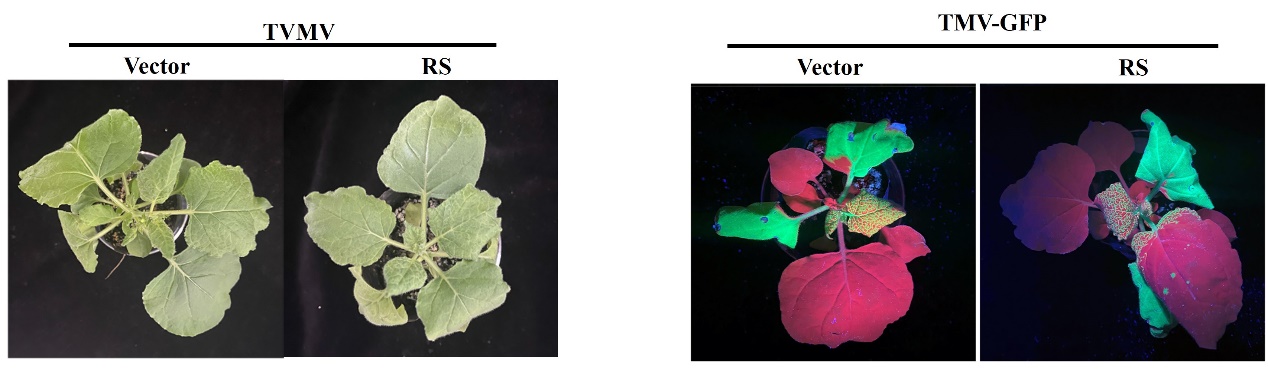
**
