# Supplementary material for: Resveratrol synthase homologs participate in infection of Nicotiana benthamiana by pathogenic plant viruses and fungi
Source: Front Microbiol. 2025 Apr 2;16:1534785. doi: 10.3389/fmicb.2025.1534785 (PMC11999985; doi:10.3389/fmicb.2025.1534785)
Supplement: Supplementary file 2 [file Table_1.docx]

**Table S1.** Primers used in this study.

|  | | |
| --- | --- | --- |
| ID | Sequence(5’-3’) | Use |
| RS-F1 | GGGGACAAGTTTGTACAAAAAAGCAGGC  TTCATGGTGTCTGTGAGTGGAATTC | Cloning |
| RS-R1 | GGGGACCACTTTGTACAAGAAAGCTGG  GTCTTATATGGCCATGCTGCGG |  |
| qPCR-RS-F2 | AAGGTCCAGCAACGGTATTG | RT-qPCR |
| qPCR-RS-R2 | AACGACGGTGCCTTATATGC |  |
| NtCOI1-F | TGTTTCTTGCTGCAAAGGTG |  |
| NtCOI1-R | GCACCTTGTTCATCCTCCAT |  |
| NtNPR1-F | TAGCGTATTGCGATGCAAAG |  |
| NtNPR1-R | TTCCATCGGATGTCAGATCA |  |
| NtLOX1-F | GCCTATGCAGCAGTGAATGA |  |
| NtLOX1-R | ATCGTCTCACGGAAATGAGG |  |
| NtAOS-F | CCACTGCTGTGCTAAACGAA |  |
| NtAOS-R | GATTTCATCAGCGGCATTTT |  |
| NtICS-F | GTTGCCTGCAGTTTCTAGCC |  |
| NtICS-R | TTCTGTGCATGAAGCCACTC |  |
| NtPAL-F | TTTGGTGCCATTGTCCTACA |  |
| NtPAL-R | ACCATAGAAGCCATGCCAGA |  |
| NtUB1-F | TCCAGGACAAGGAGGGTATCC |  |
| NtUB1-R | GTCAGCCAAGGTCCTTCCATCC |  |

**Table S2.** Amino acid composition of RS proteins.

| Amino acid | RS | |
| --- | --- | --- |
|  | No. of residues | Percentage of residues |
| Ala (A) | 29 | 7.5% |
| Arg (R) | 20 | 5.1% |
| Asn (N) | 17 | 4.4% |
| Asp (D) | 22 | 5.7% |
| Cys (C) | 7 | 1.8% |
| Gln (Q) | 11 | 2.8% |
| Glu (E) | 23 | 5.9% |
| Gly (G) | 33 | 8.5% |
| His (H) | 6 | 1.5% |
| Ile (I) | 25 | 6.4% |
| Leu (L) | 33 | 8.5% |
| Lys (K) | 23 | 5.9% |
| Met (M) | 15 | 3.9% |
| Phe (F) | 15 | 3.9% |
| Pro (P) | 20 | 5.1% |
| Ser (S) | 23 | 5.9% |
| Thr (T) | 24 | 6.2% |
| Trp (W) | 4 | 1.0% |
| Tyr (Y) | 11 | 7.2% |
| Val (V) | 28 | 9.4% |

**Table S3.** Area of necrotic spots on leaves infected by *B. cinerea*

|  | 3d | 6d | 9d | 12d |
| --- | --- | --- | --- | --- |
| Vector | 0.119 | 0.954 | 1.679 | 3.819 |
|  | 0.183 | 0.746 | 1.909 | 3.30125 |
|  | 0.151 | 0.85 | 1.794 | 2.7835 |
| RS | 0.059 | 0.508 | 0.903 | 2.062 |
|  | 0.18 | 0.466 | 0.962 | 1.45 |
|  | 0.1195 | 0.487 | 0.9325 | 1.756 |
